# Prevalence of chromosomal abnormalities and polymorphisms in 4,672 infertile patients undergoing assisted reproductive techniques in the United Arab Emirates population

**DOI:** 10.3389/frph.2026.1750389

**Published:** 2026-02-05

**Authors:** Divyesh Upadhyay, Merlin Mary Varghese, Sudha Anandt, Firas Albuz, Rawan Almekosh, Braulio Peramo

**Affiliations:** 1 Clinical Department, Al Ain Fertility Center, Al Ain, Abu Dhabi, United Arab Emirates; 2 Genetics Department, Al Ain Fertility Center, Al Ain, Abu Dhabi, United Arab Emirates; 3 IVF Department, Al Ain Fertility Center, Al Ain, Abu Dhabi, United Arab Emirates; 4 Adjunct Faculty Assistant Professor, Khalifa University, Abu Dhabi, United Arab Emirates

**Keywords:** assisted reproductive techniques (ART), chromosomal abnormalities, chromosomal polymorphisms, infertility, karyotyping

## Abstract

**Introduction:**

Chromosomal abnormalities (CA) are a key genetic contributor to infertility, particularly in regions with high consanguinity. Despite growing utilization of assisted reproductive techniques (ART) in the Gulf region, large-scale cytogenetic data remain scarce. This study aimed to determine the prevalence and distribution of CA and chromosomal polymorphisms (CP) among infertile patients undergoing ART in the United Arab Emirates (UAE), providing region-specific evidence to support diagnostic decision-making and genetic counseling.

**Methods:**

A retrospective cohort analysis was performed on 4,672 infertile patients (2,193 males and 2,479 females) who underwent conventional G-banded karyotyping between 2016 and 2024 at Al Ain Fertility Center. Semen parameters for all male participants were evaluated according to World Health Organization (WHO) standards. Cytogenetic findings were categorized into numerical abnormalities, structural abnormalities, and CP. Data were stratified by gender, infertility type, semen phenotype, and marital consanguinity (couple-level).

**Results:**

A total of 305 patients (6.5%) showed CA or CP. The prevalence of abnormalities was higher in males, with sex-chromosome aneuploidies and autosomal structural rearrangements more frequently observed among azoospermic and severely oligozoospermic men. Consanguinity (marital relatedness) was descriptively compared across couple-level karyotype groups. The proportion of consanguineous couples was 39% (644/1,639) in the normal karyotype group, 34.6% (8/24) among couples with CA, and 45.1% (40/88) among couples with CP, with no statistically significant differences between groups. These findings reinforce the diagnostic value of karyotyping, particularly in males with severe sperm abnormalities.

**Discussion/conclusion:**

This study represents the largest cytogenetic dataset on infertile patients in the UAE and the wider Gulf region, offering population-specific insights into chromosomal determinants of infertility. Routine karyotyping especially for azoospermic men remains essential for accurate diagnosis, informed counseling, and optimized ART planning. These findings provide UAE-specific prevalence data and support risk-stratified counseling; however, the consanguinity analysis reflects marital consanguinity within couples and does not assess parental consanguinity or causality.

## 1 Introduction

Infertility is a complex global health concern, with the World Health Organization (WHO) estimating that approximately 17.5% of adults, or nearly one in six individuals, will experience infertility at some point in their lives (1). The burden of infertility is distributed evenly across low-, middle-, and high-income countries, underscoring its multifactorial etiology and universal relevance. While environmental, endocrine, anatomical, and infectious factors are commonly implicated, genetic causes, particularly chromosomal abnormalities (CA), remain among the most critical contributors, especially in cases of unexplained infertility and repeated assisted reproductive technique (ART) failure.

CA includes both numerical anomalies (such as aneuploidies) and structural rearrangements (including translocations, inversions, deletions, and duplications). These abnormalities can disrupt normal gametogenesis, embryo development, and implantation, leading to infertility, recurrent pregnancy loss (RPL), or ART failure. Globally, such CA are estimated to account for approximately 10%–15% of infertility cases, with a disproportionately high prevalence among males (2). Consequently, international guidelines from the American Society for Reproductive Medicine (ASRM), the European Society of Human Reproduction and Embryology (ESHRE), and the WHO, consistently advocate cytogenetic evaluation, typically by high-resolution G- banded karyotyping, as a foundational investigation for infertile couples, especially those presenting with azoospermia, severe oligozoospermia, premature ovarian insufficiency, or RPL (3–5).

Sex chromosome aneuploidies are among the most well-established cytogenetic contributors to infertility. In males, Klinefelter syndrome (47,XXY) is the most common sex chromosome abnormality and is strongly associated with non-obstructive azoospermia, Sertoli cell-only syndrome, and impaired spermatogenesis; approximately 6% of infertile men are reported to harbor a chromosomal anomaly, with Klinefelter syndrome being the predominant finding (3). In females, Turner syndrome (45,X) and mosaic variants are closely linked to primary ovarian insufficiency, amenorrhea, and premature menopause, while structural abnormalities of the X chromosome, including deletions and X-autosome translocations, may result in infertility or recurrent miscarriage (6). Balanced structural chromosomal rearrangements, such as reciprocal and Robertsonian translocations, represent another clinically significant category. Although carriers are typically phenotypically normal, these rearrangements can generate genetically unbalanced gametes, increasing the risk of RPL, implantation failure, and embryonic aneuploidy (4, 6, 7). Their identification has direct clinical implications, including the use of preimplantation genetic testing for structural rearrangements (PGT-SR), as supported by clinical evidence, or alternative ART strategies (4, 7, 8).

Additional abnormalities, including mosaicism, marker chromosomes, deletions, and inversions, further contribute to reproductive dysfunction. Mosaicism can result in variable reproductive phenotypes depending on tissue involvement, while Y-chromosome microdeletions within the azoospermia factor (AZF) regions are well-recognized causes of male infertility (9, 10). Within the Arab region, recent evidence provides essential context regarding the prevalence of these microdeletions. A scoping review of Arab studies conducted between 2014 and 2024 revealed an overall Y-chromosome microdeletion (YCMD) frequency of 10.3% among azoospermic males, with the AZFc deletion identified as the most common subtype (11). In the UAE, a 10-year retrospective analysis of Emirati infertile men reported CA in approximately 8% of cases, with the prevalence increasing to 13.9% among azoospermic individuals (12). Furthermore, targeted diagnostic screening of Emirati azoospermic males found an AZF microdeletion frequency of 2.2%, primarily involving complete deletions in the AZFc region (13). In females, deletions involving regions such as Xp22.33 have been associated with premature ovarian insufficiency, and certain pericentric inversions may be linked to infertility or miscarriage depending on their extent (10).

Alongside these established abnormalities, chromosomal polymorphisms (CP) constitute a frequently encountered finding in infertility cytogenetics. These variants typically involve heterochromatic regions of chromosomes 1, 9, 16, and Y [e.g., 1qh+, inv(9), 16qh+, Yqh] and have traditionally been considered benign heteromorphisms. However, several studies have reported a higher prevalence of such variants among infertile individuals and couples undergoing ART, suggesting a possible subclinical association with reproductive impairment (14). Polymorphisms such as inv(9) and heterochromatic elongations are commonly observed, although their biological impact remains unclear (2, 14, 15).

Evidence regarding the clinical relevance of CP remains inconsistent. Although some studies have reported modest associations with adverse reproductive outcomes, others, including a large retrospective cohort comprising 942 ART cycles, have found no significant differences in cytogenetic or ART outcomes between polymorphism carriers and individuals with normal karyotypes. Taken together, the available literature presents mixed findings. Accordingly, CP are generally considered incidental cytogenetic variants with limited pathogenic significance; however, their identification remains clinically relevant for accurate karyotype interpretation and genetic counseling, particularly to prevent misclassification of inherited heteromorphisms during infertility evaluation, embryo assessment, or prenatal diagnosis (14, 16, 17)*.*

Despite global advances in cytogenetic profiling of infertility, there remains a significant evidence gap in the Middle East and North Africa (MENA) region. This gap is particularly relevant given the region's high prevalence of male-factor infertility, frequent consanguineous marriages, and growing reliance on ART. Studies suggest that 22.6% of men in MENA may be affected by infertility, higher than global estimates (18). Genetic contributors such as Y-chromosome microdeletions, balanced translocations, and rare autosomal variants may be amplified by consanguinity, yet few large-scale studies have systematically explored these associations in depth.

Existing cytogenetic investigations within the UAE have been limited in scope, often constrained by small sample sizes or narrow analytical focus. A recent retrospective study by Ebrahim & Mahasneh 2022 (12), reported a spectrum of karyotypic abnormalities among infertile Emirati men, including cases of Klinefelter syndrome, 45,X/46,XY mosaicism, and Robertsonian translocations. Similarly, studies from neighboring countries such as Qatar and Saudi Arabia reported CA rates ranging from 5% to 10%, particularly among individuals with azoospermia (9). However, these prior investigations did not incorporate comprehensive stratification by gender, infertility type, consanguinity status, semen profile, or chromosomal subtype within a large, representative cohort.

To address these limitations, the present study provides the first large-scale cytogenetic analysis of 4,672 individuals undergoing ART evaluation in the UAE, all of whom are members of the local Emirati population. To our knowledge, this represents the largest single-center karyotyping study in the MENA region to examine both CA and CP in a fertility-focused cohort. Importantly, the study includes extensive stratification by sex, infertility type (primary vs. secondary), and consanguinity, while also integrating detailed semen profile data across individuals with normal, polymorphic, and aberrant karyotypes. The study further distinguishes CA into subtypes such as mosaicism, marker chromosomes, and structural rearrangements, and situates UAE-specific trends within a broader global cytogenetic context.

This in-depth analysis not only fills a critical knowledge gap but also enhances the clinical relevance of cytogenetic screening and counseling in ART settings. The findings have the potential to guide personalized fertility care and improve diagnostic practices in populations with unique genetic backgrounds and cultural contexts, such as the UAE.

This study aims to provide UAE-specific data on the prevalence and patterns of CA and CP among 4,672 infertile patients undergoing ART. We determined the frequency and spectrum of CA/CP and described their distribution after stratification by sex, infertility type (primary vs. secondary), semen phenotype, and marital consanguinity. We then assessed sex differences in CA subtypes (e.g., sex chromosome anomalies, mosaicism, marker chromosomes) and evaluated statistically significant gender-specific trends. Marital consanguinity (consanguineous vs. non-consanguineous couples) was described across couple-level karyotype groups (normal, CA, CP) without inferring causality. We also compared semen analysis patterns across men with normal karyotypes, CA, and CP, focusing on azoospermia and other abnormalities, and contrasted UAE trends with international datasets to identify regionally distinct features relevant to counseling and fertility care.

## 2 Materials and methods

### 2.1 Study design and population

#### 2.1.1 Study setting and patient recruitment

This retrospective cytogenetic study was conducted at the Cytogenetic Unit, Department of Genetics, Al Ain Fertility Center, Al Ain, Abu Dhabi, United Arab Emirates. A total of 4,672 patients undergoing ART between October 2016 and December 2024 were included. All patients were referred to cytogenetic evaluation as part of their infertility workup. The cohort comprised 2,479 females (53.06%) and 2,193 males (46.94%) (Tables 1, 2). The study cohort predominantly comprised United Arab Emirates (UAE) nationals, accounting for more than 95% of the total population. The remaining cases represented non-UAE residents who received fertility care at the same tertiary referral center.

**Table 1:** Classification of studied cases based on analysis parameters.

Group	Analysis Parameter	Analysis Combination
Analysis Approach 1: Gender-Specific Analysis	**Age (SD)** – Mean ± Standard Deviation (SD)	Karyotype Result incidences with Age
*n* = 4,672		
Male: 2,193 cases	**Karyotype Result incidences** – Normal Karyotype, – Chromosomal abnormality (Category 1 & 2), – - Chromosomal Polymorphism	Karyotype Result incidences with Infertility type
Female: 2,479 cases		
	**Infertility type** – Primary Infertility – Secondary Infertility	
	**Semen analysis interpretation** – Normospermia; Teratospermia; Asthenospermia; Oligospermia; Asthenospermia + Teratospermia; Oligospermia + Teratospermia; OAT (Oligoasthenoteratospermia); SOAT (Severe OAT); Azoospermia; Semen Analysis Not Done	Karyotype Result incidences with Semen Analysis
Analysis Approach 2: Couple-Specific Analysis 1,848 couples	**Consanguinity (marital)** – Consanguineous – Non-Consanguineous Couples	Karyotype Result incidences with Consanguinity (marital)
(*n* = 3,696 individuals)		

**1. Karyotype Result Incidences** includes normal karyotype, chromosomal abnormalities, and polymorphisms.

**2. Infertility Type**: Primary (never conceived) and Secondary (previous conception but now infertile).

**3. Semen Analysis** follows WHO guidelines, including normospermia, oligospermia, asthenospermia, teratospermia, OAT (combined defects), SOAT, and azoospermia.

**4. Consanguinity (marital):** Couples were classified as consanguineous (blood relatives) or non-consanguineous, based on partner relatedness; parental consanguinity of each patient was not assessed.

**5. Total Cases**: 4,672 individual cases; couple-specific analysis includes 1,848 couples (3,696 cases).

**Table 2:** General features of the studied population.

Features	Number of patients	Number of Women	Number of Men	Age of Women	Age of Men
	(Incidence)^1^	(Incidence)^1^^,^^2^	(Incidence)^1^^,^^2^	(Mean, SD)*	(Mean, SD)*
Overall Cases	4,672	2,479 (53.06%)^1^	2,193 (46.94%)^1^	34.66 ± 7.08	36.82 ± 8.77
Normal Karyotype	4,367 (93.47%)^1^	2,330 (93.99%)^2^	2,037 (92.89%)^2^	34.66 ± 7.05	36.87 ± 8.83
Chromosomal Abnormalities	81 (1.73%)^1^	32 (1.29%)^2^	49 (2.23%)^2^	35.18 ± 6.61	35.10 ± 7.25
Chromosomal Polymorphism	224 (4.79%)^1^	117 (4.72%)^2^	107 (4.88%)^2^	34.57 ± 7.86	36.61 ± 8.28

^1^ Denotes the total number of cases analyzed in the study (***n*** **=** **4,672**), used as the denominator for all overall percentage calculations.

^2^ Refers to the total number of female and male participants: **Women (*n*** **=** **2,479)** and **Men (*n*** **=** **2,193)**; these values are used for gender-specific percentage calculations.

* Indicates that the values in the corresponding column are presented as **Mean ± Standard Deviation (SD)**.

#### 2.1.2 Inclusion criteria and clinical data collection

Eligible cases were infertile individuals or couples referred to fertility treatment, with a clinical indication for karyotyping. At the time of their initial clinical consultation, relevant demographic and clinical information, including age, gender, infertility type (primary or secondary), and marital consanguinity status, was recorded. Consanguinity was defined as marital consanguinity (blood-relatedness between the partners in the infertile couple) as recorded in clinical files. Parental consanguinity of each patient (i.e., relatedness of the patient's parents) was not available. Semen analysis for male patients was performed according to WHO guidelines, using the 5th edition manual for samples collected between 2016 and 2020 (5) and the 6th edition manual for samples analyzed from 2021 onward (19). Karyotype outcomes were classified into three main categories: normal karyotypes, CA, and CP. CA were further subtyped into two analytical categories: Category 1, comprising sex chromosome abnormalities, autosomal abnormalities, mosaicism, and marker chromosomes, and Category 2, comprising numerical, structural, or mixed CA. Individuals with normal karyotypes were designated as the control group for comparative analyses. For semen-based analyses, only cases with complete semen parameter data were included.

Analytical Stratification Criteria: To investigate the distribution and characteristics of chromosomal findings within the study population, all 4,672 patients were analyzed using two primary stratification approaches (Table 1). The first approach involved a gender-specific analysis, where all individual cases, comprising 2,193 males and 2,479 females, were assessed separately. Variables analyzed included age (reported as mean ± standard deviation), infertility type (primary or secondary), and karyotype classification (normal, CA, or CP).

The second approach focused on couple-based analysis. From the total cohort of 4,672 individuals, 1,848 couples (3,696 individuals) were identified. These couples were stratified by marital consanguinity status (consanguineous vs. non-consanguineous) to describe the distribution of couple-level karyotype groups. At the couple level, normal couples were defined as those in which both partners had normal karyotypes, whereas couples with CA or CP were defined as those in which one or both partners exhibited CA or polymorphisms, respectively. This stratification framework enabled evaluation of karyotype classifications, including normal karyotypes, CP, and CA, across both individual- and couple-based analytical units.

### 2.2 Laboratory procedures

#### 2.2.1 Karyotype sample collection, processing, and interpretation

##### 2.2.1.1 Karyotype sample collection

Fresh peripheral blood samples were collected from each patient using sterile, heparinized vacutainers under standard clinical protocols. To maintain confidentiality and traceability, each sample was labeled with a unique study code. All specimens were transported promptly to the Cytogenetic Unit at Al Ain Fertility Center and processed within 48 h of collection to preserve mitotic activity and ensure optimal metaphase quality.

##### 2.2.1.2 Karyotype sample processing (wet lab protocols)

Conventional cytogenetic processing was performed in accordance with the Standard Operating Procedure of the Al Ain Fertility Center (SOP # AAFC/LAB/P-0007) (20) and followed international technical guidelines and best practices (ACMG, 2020 (21); ACC, 2012 (22); Gardner & Sutherland, 2004 (23); AGT, 2017 (24). Peripheral blood lymphocytes were cultured using phytohemagglutinin (PHA)-stimulated media (Gibco™ PB-MAX™ Karyotyping Medium; Thermo Fisher Scientific) and incubated at 37 °C for 72 h. Colchicine was added during the final hours of incubation to arrest cells in metaphase.

Following incubation, cells underwent hypotonic treatment and fixation using methanol–acetic acid solution. G-banded metaphase chromosome spreads were then prepared using standard trypsin- Giemsa (GTG) staining protocols. The target resolution achieved was between 550 and 900 bands per haploid set, consistent with clinical diagnostic quality benchmarks outlined in the AGT Cytogenetics Laboratory Manual (24) and the Postnatal Chromosomal Analysis Guidelines (21).

##### 2.2.1.3 Karyotype analysis

Chromosome visualization and analysis were carried out using MetaSystems IKAROS software. For each case, a minimum of 20 metaphases were counted, and at least 10 were fully analyzed. In cases with suspected mosaicism or structural abnormalities, the analysis was extended to 50 to 100 metaphases to enhance diagnostic accuracy. Karyotypes were annotated and interpreted according to the International System for Human Cytogenetic Nomenclature (25).

##### 2.2.1.4 Karyotype results were classified into three primary categories

Normal karyotypes, CP, and CA. To facilitate subtype-specific evaluation, CA were further stratified into two analytical categories based on cytogenetic classification standards. Category 1 included abnormalities, such as sex chromosome abnormalities, autosomal chromosome abnormalities, mosaicism, and marker chromosomes. Category 2 comprises cases with either numerical chromosome abnormalities or structural chromosome abnormalities.

This categorization strategy was deliberately adopted to enhance analytical clarity and clinical interpretability of cytogenetic findings in the context of infertility. By distinguishing normal karyotypes, CP, and CA, and further sub-classifying abnormalities into defined analytical groups, the framework allows clear separation of benign variants from abnormalities with established or potential reproductive significance. This approach enables subtype-specific evaluation of cytogenetic patterns with differing biological mechanisms and clinical implications, facilitates standardized comparisons across sex, infertility type, consanguinity status, and semen phenotypes, and supports clinically meaningful interpretation for genetic counseling and assisted reproductive decision-making.

In addition to classical CA, the study also documented CP such as pericentric inversions and heterochromatic size variants (qh+/qh−). All findings were interpreted within a clinical context, guided by the principles outlined in Chromosome Abnormalities and Genetic Counselling by Gardner and Sutherland (23) and supported by recommendations from the Association for Clinical Cytogenetics (now integrated into the British Society for Genetic Medicine, BSGM) (22), and the American College of Medical Genetics and Genomics (ACMG) (21).

#### 2.2.2 Semen sample collection, processing, and interpretation

##### 2.2.2.1 Semen sample collection

Semen samples were obtained from male patients under standardized clinical conditions, following a period of 2 to 7 days of sexual abstinence in accordance with WHO recommendations (5, 19) Patients were instructed to collect samples in private, designated rooms using sterile, wide-mouthed, non-toxic containers. Each sample was assigned a unique study identification code to maintain traceability and ensure patient confidentiality. Specimens were immediately delivered to the laboratory to prevent degradation and processed without delay. All procedures adhered to the institutional guidelines described in the Standard Operating Procedure of the Al Ain Fertility Center (SOP # AAFC/IVF/SOP-060) (20).

##### 2.2.2.2 Semen sample processing (wet lab protocols)

Upon arrival at the laboratory, the semen samples were first allowed to liquefy at room temperature. Standardized macroscopic and microscopic evaluations were then performed. Macroscopic assessments included semen volume and pH, while microscopic evaluation comprised sperm concentration, total and progressive motility, vitality, and morphology, assessed according to Kruger's strict criteria. Additional parameters such as the presence of leukocytes and cellular debris were also documented. All wet lab procedures were conducted in strict accordance with WHO laboratory guidelines, applying the 5th edition manual (2010) for samples collected between 2016 and 2020 and the 6th edition manual (2021) for samples processed from 2021 onward (5, 19). Throughout all stages of sample handling and analysis, rigorous quality control measures were implemented to ensure consistency and reproducibility of results across technicians and over time.

##### 2.2.2.3 Semen analysis

Following processing, semen parameters were interpreted using WHO reference values to categorize each sample as either normozoospermic or abnormal. Abnormal profiles were further classified based on deviations in sperm count, motility, and morphology, using standardized diagnostic thresholds provided in the respective WHO manuals. The final semen profile interpretations included categories such as oligozoospermia, asthenozoospermia, teratozoospermia, oligoasthenoteratozoospermia (OAT), severe oligoasthenoteratozoospermia (SOAT), and azoospermia.

### 2.3 Statistical analysis

All statistical analyses were conducted using GraphPad Prism version 9.5.0 (26). Descriptive statistics were applied to summarize continuous variables such as age, which was reported as mean ± standard deviation (SD) or median with range, where appropriate. The period prevalence of CA and polymorphisms was calculated relative to the total cohort (*n* = 4,672).

Comparative analyses were performed to assess associations between chromosomal classifications (normal, CP, CA) and demographic or clinical parameters such as gender, consanguinity, and semen characteristics. Chi-square tests were used for categorical comparisons when expected frequencies met standard assumptions; otherwise, Fisher's exact test was employed. Statistically significant results were defined as those with a two-tailed *P*-value of ≤ 0.05. The significance levels are represented as: *P* ≤ 0.05 (*): Statistically significant; *P* ≤ 0.01 (**): Moderately significant; *P* ≤ 0.001 (***): Highly significant; *P* ≤ 0.0001 (****): Very highly significant, while *P* > 0.05 was considered non- significant (NS). The statistical test applied, and exact *P*-values are documented in each respective result table.

### 2.4 Ethical considerations

All karyotyping and semen analyses were performed as part of routine clinical care, for which written informed consent was obtained from all patients at the time of testing. Given the retrospective nature of the study and the use of fully anonymized data, the requirement for additional study-specific informed consent was waived by the Al Ain Fertility Center Research Ethics Committee, Abu Dhabi, U.A.E. (Project ID: AAFC/CREC/002). Confidentiality of patient records and cytogenetic data was strictly maintained in accordance with institutional and ethical guidelines.

## 3 Results

### 3.1 Study population overview

A total of 4,672 infertile patients undergoing ART were included in the analysis, with a balanced gender distribution and comparable age profiles between males and females (Tables 1, 2). Overall karyotype distribution revealed that most patients exhibited normal karyotypes, while CA and CP were identified in smaller proportions, as detailed in Table 2 and Supplementary Tables S1–S2.

Gender-specific differences in karyotype outcomes were observed across the cohort. The detailed sex-based distribution of normal karyotypes, CA, and CP is presented in the subsequent section and summarized in Table 2.

Evaluation of infertility type revealed that secondary infertility was more prevalent across the cohort, reported in 3,179 cases (72.8%, 3,179/4,367), compared to primary infertility in 1,188 cases (27.2%, 1,188/4,367) among patients with normal karyotypes (Supplementary Table S2). Among men with normal karyotypes, 839 (41.2%) had primary infertility and 1,198 (58.8%) had secondary infertility.

In women, 349 (15.0%) had primary and 1,981 (85.0%) had secondary infertility.

Regarding consanguinity, among the 1,639 couples with normal karyotypes (3,278 individuals), 61.0% (995/1,639) were non-consanguineous and 39.0% (644/1,639) were consanguineous. Among couples with CA (*n* = 24), 65.4% (16/24) were non-consanguineous and 34.6% (8/24) consanguineous. Similarly, for the CP group (*n* = 88 couples), 54.9% (48/88) were non-consanguineous and 45.1% (40/88) consanguineous. Statistical comparisons showed no statistically significant differences in the distribution of marital consanguinity between couples with normal karyotypes and those with CA (*P* = 0.5528) or CP (*P* = 0.2495). (Table 3; Supplementary Table S1).

**Table 3:** Distribution of infertility patients based on consanguinity in the control and studied groups.

Features	Control Group^1^	Studied Group^4^			
	^5^Consanguinity (marital) in Couples with Normal Karyotype (Individuals)^1^	^5^Consanguinity (marital) in Couples with Chromosomal Abnormalities (Individuals)^2^	*p*-value Normal vs. Chromosomal Abnormalities	^5^Consanguinity (marital) in Couples with Chromosomal Polymorphism (Individuals)^3^	*p*-value Normal vs. Chromosomal Polymorphism
Non-Consanguineous	995 (61%)	16 (65.4%)	**0.5528**	48 (54.9%)	**0.2495**
Consanguineous	644 (39%)	8 (34.6%)		40 (45.1%)	
Overall Cases	**1,639** (3,278 Individuals)	**24** (48 Cases)		**88** (176 Cases)	

The Chi-square test (*χ*^2^) was used for all parameters in this table to compare the distribution of consanguinity in the control and studied groups. Significance levels are represented as follows: *P* ≤ 0.05, *P* ≤ 0.01, *P* ≤ 0.001, *P* ≤ 0.0001. Statistically significant values are bolded. Results with *P* > 0.05 are considered not significant (NS).

^1^ **Couples with Normal karyotypes**: Both partners have normal karyotypes (Control Group).

^2^ **Couples with Chromosomal Polymorphism**: One or both partners have chromosomal polymorphism, with at least one being normal.

^3^ **Couples with Chromosomal Abnormality**: One or both partners have chromosomal abnormalities.

^4^ **Studied Group:** Includes infertile couples with Chromosomal polymorphism and Chromosomal Abnormalities.

^5^ **Consanguinity (marital):** Consanguinity refers to partner-relatedness within the infertile couple (marital consanguinity); parental consanguinity data were not available.

### 3.2 Karyotype result distribution

Among the 4,672 patients evaluated, normal karyotypes were observed in the majority of cases, while CA and CP were detected in smaller proportions, as summarized in Table 2. The specific distribution of these abnormality subtypes is presented in Table 4.

**Table 4:** Frequency of chromosomal abnormalities (category 1 & 2) and their subclassification in infertility patients.

Chromosome Abnormality (Category 1) Subtypes	Males (*n* = 49)	Incidence (*n*/49, %)	Females (*n* = 32)	Incidence (*n*/32, %)	Grand Total (*n* = 81)	Grand Total Incidence (*n*/81, %)	Statistically Significance *P* value (*P* < 0.05)
Sex Chromosome abnormalities	**31**	63.27%	**4**	12.50%	**35**	43.21%	**<0** **.** **0001** ****
Autosomal Chromosome abnormalities	**13**	26.53%	**13**	40.63%	**26**	32.09%	0.1841
Mosaicism	**4**	8.16%	**13**	40.63%	**17**	20.99%	**0** **.** **0005** ***
Marker chromosome abnormalities	**-**	-	**2**	6.25%	**2**	2.47%	0.1531
Other: Autosomal + Sex Chromosome abnormalities	**1**	2.04%	**0**	0	**1**	1.23%	>0.9999
Chromosome Abnormality (Category 2) Subtypes	Males (*n* = 49)	Incidence (*n*/49, %)	Females (*n* = 32)	Incidence (*n*/32, %)	Grand Total (*n* = 81)	Grand Total Incidence (*n*/81, %)	Statistically Significance *P* value (*P* < 0.05)
Numerical chromosome abnormalities	**33**	67.35%	**16**	50.00%	**49**	60.49%	0.1185
Structural chromosome abnormalities	**15**	30.61%	**15**	46.88%	**30**	37.04%	0.1384
Combined: Structural + Numerical chromosome abnormalities	**0**	-	**1**	3.13%	**1**	1.23%	0.3951
Unclassified: Not Structural Or Numerical chromosome abnormalities	**1**	2.04%	**0**	-	**1**	1.23%	>0.9999

The Chi-square test (*χ*^2^) was used to compare chromosomal abnormality subtypes between males (*n* = 49) and females (*n* = 32) for variables where all expected cell frequencies were ≥ 5, including Sex Chromosome Abnormalities, Autosomal Chromosome Abnormalities, Mosaicism, Numerical and Structural Chromosome Abnormalities. For subtypes with low cell counts, such as Marker Chromosome Abnormalities, Combined (Structural + Numerical), and Other (Autosomal + Sex), Fisher's exact test was applied. The significance levels are represented as:

* *P* ≤ 0.05: Statistically significant.

** *P* ≤ 0.01: Moderately significant.

*** *P* ≤ 0.001: Highly significant.

**** *P* ≤ 0.0001: Very highly significant.

Statistically significant values are **bolded**. Results with P > 0.05 are considered not significant (NS).

Gender-specific analysis showed that normal karyotypes were slightly more prevalent in females (93.99%, 2,330/2,479) than in males (92.89%, 2,037/2,193). CA were more frequently observed among males (2.23%, 49/2,193) compared to females (1.29%, 32/2,479). In contrast, CP occurred at comparable frequencies in males (4.88%, 107/2,193) and females (4.72%, 117/2,479) (Table 4).

### 3.3 Chromosomal abnormalities (category 1 & 2) subtype breakdown

As summarized in Table 4 the 81 patients identified with chromosomal abnormalities (CA) were analyzed using two complementary classification frameworks to provide a comprehensive characterization of the findings.

Under the Category 1 classification (which categorizes abnormalities by specific chromosomal involvement or the presence of mosaicism), sex chromosome abnormalities were the most frequent subtype, accounting for 35 cases (43.21%, 35/81). These abnormalities exhibited a marked sex disparity, being significantly more prevalent in males (63.27%, 31/49) compared to females (12.50%, 4/32), with the association reaching statistical significance (*P* < 0.0001), as shown in Table 3. Autosomal abnormalities were detected in 26 patients (32.10%, 26/81), with a relatively balanced distribution between females (40.63%, 13/32) and males (26.53%, 13/49) and no statistically significant sex difference (*P* = 0.1841). Mosaicism was observed in 17 cases (20.99%, 17/81), and was significantly more common among females (40.63%, 13/32) than males (8.16%, 4/49), with *P* = 0.0005. Marker chromosome abnormalities were rare, present in 2 patients (2.47%, 2/81), both of whom were female (*P* = 0.1531). A single case (1.23%, 1/81) exhibited a complex CA involving both autosomal and sex chromosomes; this was observed in a male and was not statistically significant (*P* > 0.9999). These subtype distributions highlight important sex-linked trends in CA patterns within the infertile population under study.

When the same 81 cases were re-evaluated under the Category 2 classification (which distinguishes abnormalities by their numerical or structural nature), numerical abnormalities were found in 49 cases (60.49%, 49/81), followed by structural abnormalities in 30 cases (37.04%, 30/81). One female (1.23%, 1/81) had a combination of structural and numerical abnormalities, and one male (1.23%, 1/81) had an unclassified anomaly (Table 4). No statistically significant sex-based differences were observed in any Category 2 subtype (*P* > 0.05 for all comparisons).

Detailed karyotype classifications including syndromic cases, such as Klinefelter syndrome (47,XXY) in 26 males, 47,XYY in 4 males, Triple X (47,XXX) in 2 females, and multiple reciprocal or Robertsonian translocations, are presented in Supplementary Tables S3–S5.

### 3.4 Chromosome polymorphism subtype breakdown

Among the 224 patients identified with CP, the majority involved autosomal chromosomes, while sex chromosome polymorphisms were notably rare and observed exclusively in males, indicating a statistically significant gender association (Table 5). Multiple polymorphisms were present in 11 patients (4.91%), with a higher incidence in females (6.84%) than in males (2.80%), although this difference did not reach statistical significance (*P* = 0.2201). Detailed cytogenetic evaluation further revealed that heterochromatic variants such as qh + were predominant among autosomal polymorphisms. Inversions were most commonly reported on chromosome 9, and satellite/stalk polymorphisms were observed across various acrocentric chromosomes. These patterns are elaborated in Supplementary Tables S6, S7.

**Table 5:** Frequency of chromosomal polymorphisms.

Features	Number of Women (Incidence)^1^^,^^2^^,^^3^	Number of Men (Incidence)^1^^,^^2^^,^^3^	Grand Total Incidence (*n*/224, %)	Statistically Significance *P* value (*P* < 0.05)
Chromosomal Polymorphism	117 (4.72%)^2^	107 (4.88%)^2^	224 (4.79%)^1^	
Autosomal chromosomes	109 (93.16%)^3^	95 (88.79%)^3^	204 (91.07%)^3^	0.2511
Multiple polymorphisms	8 (6.84%)^3^	3 (2.80%)^3^	11 (4.91%)^3^	0.2201
Sex chromosomes	0	9 (8.41%)^3^	9 (4.02%)^3^	**0** **.** **0011** **

^1^ Calculated using the total number of cases analyzed in the study (***n*** **=** **4,672**), which serves as the denominator for all overall percentage calculations.

^2^ Calculated using the total number of **women** (***n*** **=** **2,479**) and **men** (***n*** **=** **2,193**) in the study; these serve as the denominators for gender-specific incidence calculations.

^3^ Percentages for chromosomal polymorphism subtypes are calculated based on the number of patients with polymorphisms in each group: • Overall: ***n*** **=** **224**, Women: ***n*** **=** **117**, Men: ***n*** **=** **107.**

The Chi-square test (*χ*^2^) was used for Autosomal Chromosome Polymorphisms. Fisher's exact test was applied to Multiple Polymorphisms and Sex Chromosome Polymorphisms. The significance levels are represented as:

* *P* ≤ 0.05: Statistically significant.

** *P* ≤ 0.01: Moderately significant.

*** *P* ≤ 0.001: Highly significant.

**** *P* ≤ 0.0001: Very highly significant.

Statistically significant values are bolded. Results with *P* > 0.05 are considered not significant (NS).

### 3.5 Cytogenetic findings by infertility type (primary & secondary infertility)

#### 3.5.1 Chromosomal abnormalities (category 1)

Among the 81 patients identified with Category 1 CA (1.73%, 81/4,672), 47 cases (58.0%, 47/81) were associated with primary infertility, while 34 cases (42.0%, 34/81) had secondary infertility (Supplementary Table S3). When stratified by sex, a significant proportion of males (73.5%, 36/49) with abnormalities were affected by primary infertility, in contrast to 34.4% (11/32) of females. Conversely, secondary infertility was more commonly reported among females (65.6%, 21/32) compared to males (26.5%, 13/49).

Further subtype analysis from Supplementary Table S4 showed that sex chromosome abnormalities were predominantly linked to primary infertility, accounting for 88.6% (31/35) of such cases, with the majority occurring in males (27/31). In contrast, autosomal abnormalities demonstrated a more balanced distribution, with 30.8% (8/26) associated with primary infertility and 69.2% (18/26) with secondary infertility. Mosaicism, observed in 17 individuals (predominantly females), was slightly more frequent among those with secondary infertility (64.7%, 11/17) compared to primary (35.3%, 6/17). Rare abnormalities such as marker chromosome abnormalities and combined autosomal + sex chromosome anomalies were detected in only one to two individuals and were primarily linked to primary infertility. These findings, detailed in Supplementary Table S3, suggest a strong association between certain abnormality subtypes, particularly sex chromosome anomalies, and the etiology of primary infertility, especially in males.

#### 3.5.2 Chromosomal abnormalities (category 2)

Of the 81 patients with Category 2 CA, 60.5% (49/81) were numerical abnormalities and 37.0% (30/81) were structural, with one case each of combined and unclassified abnormalities (Table 3).

Among those with numerical abnormalities, primary infertility was reported in 67.3% (33/49) of cases, with a notable male predominance (28/33). Structural abnormalities were somewhat evenly distributed between primary infertility (12/30) and secondary infertility (18/30).

The unclassified and combined structural + numerical categories (each *n* = 1) were exclusively observed in cases of primary infertility (Supplementary Table S5).

#### 3.5.3 Chromosomal polymorphism

Out of the 224 patients with CP (4.79%, 224/4,672), 101 individuals (45.1%) were diagnosed with primary infertility, while 123 patients (54.9%) presented with secondary infertility (Supplementary Table S2). When analyzed by sex, 50.5% (54/107) of affected males had primary infertility, compared to 40.2% (47/117) of females, indicating a mild male predominance in the primary infertility group among polymorphic carriers.

Subtype-specific analysis from Supplementary Table S5 revealed that autosomal polymorphisms were the most prevalent subtype, accounting for 91.1% (204/224) of all polymorphism cases. These were evenly distributed across infertility types, with 101 cases in primary infertility and 123 in secondary infertility. Notably, sex chromosome polymorphisms (*n* = 9) were detected exclusively in males, and all occurred in males with primary infertility, suggesting a possible sex-linked cytogenetic influence in this group. Multiple polymorphisms, although rare (*n* = 11), showed a higher occurrence in primary infertility (72.7%, 8/11) compared to secondary infertility (27.3%, 3/11). These trends highlight the predominance of autosomal variants and raise the potential clinical relevance of sex chromosome polymorphisms in primary male infertility.

### 3.6 Cytogenetic findings by consanguinity in the control and studied groups

Analysis of consanguinity status was conducted across three groups: couples with normal karyotypes (control group), couples with CA, and couples with CP (Table 3). Among the 1,639 couples with normal karyotypes (3,278 individuals), 995 couples (61.0%) were non-consanguineous, and 644 couples (39.0%) were consanguineous. In the group with CA (*n* = 24 couples, 48 individuals), 16 couples (65.4%) were non-consanguineous, and 8 couples (34.6%) were consanguineous. Similarly, in the CP group (*n* = 88 couples, 176 individuals), 48 couples (54.9%) were non-consanguineous .and 40 couples (45.1%) were consanguineous.

Statistical comparison using the Chi-square test showed no significant differences in the proportion of consanguineous vs. non-consanguineous couples between the control group and those with CA (*P* = 0.5528), or between the control group and those with CP (*P* = 0.2495) (as shown in Table 3). Thus, consanguinity was not statistically associated with the presence of CA or polymorphisms in this cohort.

### 3.7 Cytogenetic findings by semen analysis patterns

Overall semen phenotype distribution (entire infertile male cohort): Semen analysis was available for 2,181 of 2,193 infertile males (12 cases had no semen analysis, all within the polymorphism group). In the overall male cohort with available semen analysis (*n* = 2,181), the most frequent semen phenotype was teratospermia (53.42%, 1,165/2,181), followed by normospermia (22.65%, 494/2,181) and SOAT; 9.35%, 204/2,181. Combined abnormalities were also observed, including OAT; 4.63%, 101/2,181, asthenospermia + teratospermia (4.49%, 98/2,181), and oligospermia + teratospermia (3.94%, 86/2,181). Azoospermia represented 1.42% (31/2,181) of the overall infertile male cohort, while isolated oligospermia and asthenospermia were rare (0.05% each; 1/2,181) [Table 6].

**Table 6:** Overall and cytogenetic subgroup distribution of semen abnormalities in infertile males.

Semen Parameters	Overall Infertile Male Population (Incidence)^0^ (*n* = 2,181), *n* (%)	Normal Cases (Incidence)^1^ (*n*/2,037, %)	Chromosomal Abnormalities Cases (Incidence)^2^ (*n*/49, %)	*p*-value	Chromosomal Polymorphism Cases (Incidence)^3^ (*n*/95, %)	*p*-value
				Normal vs. Chromosomal Abnormalities		Normal vs. Chromosomal Polymorphism
Teratospermia	1,165 (53.42%)	1,121 (55.03%)	-	-	44 (46.32%)	0.0,953
NORMOSPERMIA	494 (22.65%)	460 (22.58%)	11 (22.45)	0.9,824	23 (24.21)	0.7,109
SOAT	204 (9.35%)	188 (9.23%)	4 (8.16)	>0.9,999	12 (12.63)	0.2,776
Asthenospermia + Teratospermia	98 (4.49%)	96 (4.71%)	-	-	2 (2.11)	0.3,188
OAT	101 (4.63%)	91 (4.47%)	5 (10.20)	0.0,716	5 (5.26)	0.6,146
Oligospermia + Teratospermia	86 (3.94%)	79 (3.88%)	-	-	7 (7.37)	0.1,032
Oligospermia	1 (0.05%)	1 (0.05%)	-	-	-	-
Asthenospermia	1 (0.05%)	1 (0.05%)	-	-	-	-
Azoospermia	31 (1.42%)	0	29 (59.18)	**<0.0,001** **** ^,^ ^a^	2 (2.11)	**0.002** *** ^,^ ^a^
Semen Analysis Not Done (*n* = 12)	-	-	-	-	-	-
Grand total	**2,181** **(100%)**	2,037	49	-	95	-

The Chi-square test (*χ*^2^) was used for Teratospermia and Normospermia, while Fisher's exact test was applied to all other parameters in this table. Values marked with:

^a^ Should be interpreted with caution, as one group had zero cases.

The significance levels are represented as:

* *P* ≤ 0.05: Statistically significant.

** *P* ≤ 0.01: Moderately significant.

*** *P* ≤ 0.001: Highly significant.

**** *P* ≤ 0.0,001: Very highly significant.

Statistically significant values are bolded. Results with *P* > 0.05 are considered not significant (NS). “**-**” indicates that statistical analysis was not performed as one group had zero cases.

^0^ **Incidence in Overall Cases**: The percentage is calculated as **(*n*/2,181)** **×** **100**, representing only the males with available semen analysis (Total males 2,193—12 cases without analysis = 2,181).

^1^ **Incidence in Normal Cases**: The percentage is calculated as **(*n*/2,037)** **×** **100**, where 2,037 represents the total number of males with a **normal karyotype**.

^2^ **Incidence in Chromosomal Abnormality Cases**: The percentage is calculated as **(*n*/49)** **×** **100**, where 49 represents the total number of males with **chromosomal abnormalities**.

^3^ **Incidence in Chromosomal Polymorphism Cases**: **(*n*/95)** **×** **100**, (107 − 12 = 95) excluding 12 cases without semen analysis.

When stratified by cytogenetic status, the normal karyotype group (*n* = 2,037) followed a similar distribution to the overall cohort, with teratospermia remaining the most prevalent phenotype at 55.03% (1,121/2,037), followed by normospermia (22.58%) and SOAT (9.23%), as detailed in Table 6.

Azoospermia showed the strongest association with CA, being present in 59.18% (29/49) of such cases. In contrast, azoospermia was not observed in the normal karyotype group (0/2,037) and was detected in only 2.11% (2/95) of males with CP. These differences were highly statistically significant in both comparisons: normal vs. abnormalities (*P* < 0.0,001****) and normal vs. polymorphisms (*P* = 0.002***) (Table 6).

Other semen abnormalities, including OAT, SOAT, and teratospermia, were observed in all cytogenetic groups but did not differ significantly. For instance, OAT was reported in 10.20% (5/49) of patients with CA compared to 4.47% (91/2,037) among those with normal karyotypes (*P* = 0.0716, NS). Normospermia was similarly distributed: 22.58% (460/2,037) in the normal group, 22.45% (11/49) in the abnormality group, and 24.21% (23/95) in the polymorphism group (*P* > 0.70, NS).

## 4 Discussion

Infertility is not merely a medical condition, it is a complex and growing public health concern influenced by complex demographic, genetic, and societal factors. In regions like the Middle East, where consanguinity rates remain high and infertility carries both clinical and social implications, cytogenetic investigations are crucial for uncovering underlying genetic contributors. Against this backdrop, our large-scale cytogenetic study offers a uniquely comprehensive demographic and genetic snapshot of infertile patients undergoing ART in the United Arab Emirates (UAE), providing valuable insights to inform region-specific reproductive health strategies.

With a balanced gender representation, 53.06% females (2,479/4,672) and 46.94% males (2,193/4,672), the study allows for an equitable interpretation of cytogenetic trends and gender- specific analyses. The mean age observed in males (36.82 ± 8.77 years) was slightly higher than in females (34.66 ± 7.08 years), a trend that aligns with regional reproductive patterns, reflecting common scenarios in ART populations where male partners often present later in the reproductive timeline (Table 2).

A notably high prevalence of normal karyotypes (93.47%, 4,367/4,672) was observed, reinforcing the role of non-cytogenetic factors in infertility for the majority of ART patients. Despite this high rate, the detection of CA (1.73%, 81/4,672) remains clinically significant, particularly given their potential impact on reproductive outcomes. Comparing this finding with global literature (Supplementary Table S8), our overall abnormality rate (1.73%) is consistent with findings from Italy (1.97%) (27), yet notably lower than those reported in Germany (15.68%) and Egypt (13.5%) (28, 29). Such variations may reflect differences in genetic backgrounds, diagnostic methods, and patient selection criteria, underscoring the importance of localized cytogenetic assessments. Furthermore, our findings align with European observations from Hungary reported by Andó et al. (8.07%) (30) and France by Butnariu et al. (8.08%) (31), where abnormality rates were similarly reported in mid-range percentages compared to the extremes seen in other regions.

Gender differences in CA were evident, with males showing a higher frequency (2.23%, 49/2,193) compared to females (1.29%, 32/2,479). This observation aligns with existing literature that documents higher male susceptibility to chromosomal anomalies, particularly sex chromosome abnormalities linked to impaired spermatogenesis, such as Klinefelter syndrome and Y-chromosome microdeletions. In the context of female infertility, region-specific cytogenetic evidence from neighboring Gulf and Arab populations also supports the clinical relevance of sex-chromosome abnormalities and mosaicism in women undergoing infertility-related evaluation. In a Saudi cohort of women referred for amenorrhea assessment, abnormal karyotypes were identified in approximately 19% of primary amenorrhea cases, with 45,X and 46,XY among the most frequently observed abnormal karyotypes, reinforcing the role of sex-chromosome errors and disorders of sex development in female reproductive dysfunction (32). Complementing this Gulf evidence, an Arab-region infertility cohort reported that among infertile females, numerical sex-chromosome abnormalities (8.72%), structural sex-chromosome abnormalities (3.6%), and X-chromosome mosaicism (2.56%) constituted a substantial proportion of cytogenetic findings, with a smaller fraction showing 46,XY (1.39%) (29). Although UAE-specific female cytogenetic series remain comparatively limited, these regional data provide an appropriate contextual framework for interpreting the female cytogenetic patterns observed in our UAE cohort and underscore the value of routine karyotyping in women presenting with infertility, amenorrhea, or recurrent pregnancy loss within the Gulf region.

CP were identified in 4.79% (224/4,672) of the study population, with a nearly equal distribution between males (4.88%) and females (4.72%), indicating no evident sex-specific predisposition (Supplementary Table S2). This prevalence is broadly consistent with international observations, being lower than the higher rates reported from India (16.7%) (15), yet exceeding those documented in Chinese cohorts (3.42% and 2.15%) (10, 33) (Supplementary Table S8). Other large-scale Chinese studies have reported varied polymorphism rates, ranging from 2.40% to as high as 7.05%, suggesting significant ethnic or methodological variability within the region (34, 35).

The absence of a marked gender bias supports the interpretation that CP, although relatively common, may have limited relevance to sex-specific infertility mechanisms.

Within the normal karyotype group, secondary infertility was predominant (72.8%, 3,179/4,367), particularly among female patients (85.0%, 1,614/1,899), a pattern that may reflect evolving demographic and clinical factors such as delayed childbearing and increased utilization of ART (Supplementary Table S2). This observation underscores the importance of addressing post-conception reproductive health challenges, including pelvic infections and miscarriage-related complications.

Consanguinity was frequently observed among couples with normal karyotypes (39.0%, 644/1,639), consistent with established regional marital practices. However, the lack of a significant association with CA or CP suggests that consanguinity may exert a limited direct influence on large-scale chromosomal alterations detectable by conventional cytogenetic analysis. This finding is relevant for genetic counseling in consanguineous populations, where monogenic disorders may represent a more substantial genetic risk than macro-level karyotype abnormalities (Table 3). Building upon this regional demographic framework, a granular evaluation of chromosomal subtypes and their comparative prevalence provides deeper insights into how these UAE-specific patterns align with international data.

This comprehensive cytogenetic analysis reveals that the vast majority of patients seeking ART exhibit normal karyotypes (93.47%, 4,367/4,672; Table 2), highlighting that chromosomal factors account for infertility in only a small subset of this population. Nevertheless, the presence of CA in 1.73% (81/4,672) and CP in 4.79% (224/4,672) of the studied cohort remains clinically significant, underscoring the necessity of routine karyotype screening in infertility assessments (Table 2).

Notably, the overall CA rate observed in this UAE cohort (1.73%) aligns closely with report from European population such as Italy (1.97%) (27), and regional data from Egypt (1.18%) (36),, yet is significantly lower than those reported in Germany (15.68%) (28),, as illustrated in Supplementary Table S8. Such variations might reflect differences in genetic backgrounds, diagnostic criteria, or environmental exposures, emphasizing the need for context-specific data to guide clinical practice in reproductive genetics.

Subtype analysis of CA (Table 4) revealed that sex chromosome abnormalities were more prevalent than autosomal abnormalities, with Klinefelter syndrome (47,XXY) being the most common subtype in males (24.49%, 12/49), and Turner mosaicism (45,X/46,XX) and other sex chromosome mosaicisms being more prevalent among females. Autosomal abnormalities (e.g., Robertsonian and reciprocal translocations) were relatively rare and evenly distributed between genders. Mosaicism, primarily involving structural variants and fragile sites, was more frequently identified in females (1.01%, 25/2,479) compared to males (0.09%, 2/2,193), with statistically significant sex-based differences (*P* = 0.0,005; Table 3).

These subtype distributions align with established UAE-specific and regional evidence (12, 37). A 10-year retrospective study of Emirati infertile men by Ebrahim & Mahasneh (2022) similarly identified Klinefelter syndrome (47,XXY) as the most frequent numerical abnormality, representing 4.0% of their total cohort and 71.4% of all numerical cases (12). Our identification of 47,XYY (Jacob syndrome) as a secondary male finding also mirrors their reported 0.8% prevalence in the Emirati population (12), as well as findings from Egypt, where Klinefelter syndrome (8.7%) was identified as the predominant genetic cause in men presenting with severe male-factor infertility (38). In females, our observation that Turner mosaicism and other sex chromosome variations predominate is consistent with Saudi Arabian data, where 45,X and its variants were among the leading chromosomal etiologies in women referred for reproductive evaluation (32). This is contrasted by higher CA frequencies reported in Saudi Arabia (12.5%) and Morocco (11.0%), highlighting how patient selection criteria and regional genetic factors influence reported prevalence (39, 40). Furthermore, large-scale Egyptian studies have reported that numerical sex chromosome abnormalities (8.72%) and mosaicism (2.56%) constitute the bulk of female cytogenetic findings, reinforcing the regional trend of sex chromosome susceptibility over autosomal rearrangements (29). While the relatively low distribution of autosomal structural abnormalities observed in our study is corroborated by wider regional data, the clinical manifestation of these genetic subtypes in semen phenotypes can vary; for instance, while we observed a strong link between CA and azoospermia, other GCC populations like Qatar have reported significantly higher baseline rates of azoospermia (6.05%) compared to the findings in our UAE-based cohort (41).

Gender-specific analyses revealed significant disparities in the distribution of CA. Males exhibited a higher rate of CA (2.23%, 49/2,193) compared to females (1.29%, 32/2,479; Table 4). This disparity is primarily attributed to well-documented syndromes such as Klinefelter syndrome (47,XXY) and other sex chromosome anomalies like 47,XYY, which have profound impacts on male fertility through mechanisms of impaired spermatogenesis. In contrast, mosaicism was predominantly identified in female cases, suggesting a different etiological pattern possibly associated with gonadal dysgenesis or embryonic rescue mechanisms.

CP were relatively evenly distributed between genders, affecting 4.88% (107/2,193) of males and 4.72% (117/2,479) of females. This nearly equal distribution supports the interpretation that most autosomal polymorphisms, such as inv(9), heterochromatic variants like 1qh+, and certain Y chromosome polymorphisms, are benign variants with limited clinical relevance. However, the exclusive presence of sex chromosome polymorphisms in males (8.41%, 9/107), particularly involving Y chromosome structural variants, suggests a potential subclinical influence on spermatogenesis that warrants further investigation (Table 4).

Further dissection of polymorphic variants (Supplementary Table S3) revealed that heterochromatic variants (qh+/qh−) accounted for the majority of cases, with chromosomes 1 and 16 being most frequently involved. Among autosomal polymorphisms, inv(9) was a recurring feature, while other structural variants such as chromosome 3 inversions and stalk-satellite configurations were comparatively rare. Interestingly, cases with multiple polymorphisms involving more than one chromosomal locus appeared more frequently in females than in males.

The comparable distribution of polymorphisms across genders and the dominance of autosomal involvement align with previous studies, although the prevalence in this study (4.79%) lies between rates reported in China (2.15%–3.42%) and India (16.7%) as detailed in Supplementary Table S8. This suggests the presence of regional and ethnic variability in polymorphism distribution, which should be considered in clinical interpretations.

The clear distinction between benign polymorphisms and clinically significant CA remains critical for appropriate reproductive counseling and ART planning. These findings reinforce the need for detailed chromosomal subtype classification (Tables 3–6) and support the implementation of sex-specific diagnostic strategies, particularly in genetically diverse populations. Beyond these general prevalence patterns and sex-specific diagnostic strategies, the clinical utility of cytogenetic screening is further refined when findings are analyzed in relation to the patient's specific reproductive history, particularly the distinction between primary and secondary infertility.

The present study identifies meaningful associations between infertility type and chromosomal findings, providing essential insights for clinical practice in reproductive medicine. Among patients with normal karyotypes (93.47%, 4,367/4,672), secondary infertility predominated at 72.8% (3,179/4,367), particularly in females (85.0%, 1,981/2,330) compared to males (58.8%, 1,198/2,037) as shown in Supplementary Table S2. This reflects regional demographic trends such as delayed childbearing, increased access to ART, and reproductive histories involving prior pregnancies or miscarriages (12). These findings highlight that non-genetic factors, such as age-related fertility decline, reproductive tract pathology, or lifestyle influences, are critical contributors to infertility in cytogenetically normal individuals (33). This is particularly relevant in the Arab world, where a significant epidemiological decline in human fertility rates has been documented over the past decade, including in the UAE where fertility rates have reached notable lows (42). In the Gulf region, this trend is further influenced by a high prevalence of reproductive diseases and various comorbidities; for instance, large-scale evidence from Qatar indicates that conditions such as polycystic ovarian syndrome (PCOS), uterine polyps, and endometriosis in females, and varicoceles and erectile dysfunction in males, are associated with significantly higher odds of infertility among both national and expatriate populations (43). These regional insights underscore that the infertility burden in the UAE is multifactorial, driven by a combination of shifting demographic trends and a high local burden of reproductive pathology (42, 43).

In contrast, primary infertility was notably more common among patients with CA. Among individuals with CA (1.73%, 81/4,672), 58.0% (47/81) had primary infertility (Supplementary Table S2), and this trend was especially prominent in males, where 73.5% (36/49) exhibited primary infertility. This pronounced link is largely driven by numerical sex chromosome anomalies such as Klinefelter syndrome (47,XXY) and 47,XYY, both of which are recognized for their deleterious effects on spermatogenesis and testicular function, Supplementary Table S4, (15). These findings reinforce the recommendation for routine karyotype screening in males with unexplained primary infertility, especially when severe oligozoospermia or azoospermia is present.

In females with CA, secondary infertility was more frequent (65.6%, 21/32; Supplementary Table S2), suggesting potential associations with structural CA or mosaicism that may not prevent initial conception but impact later reproductive outcomes (Supplementary Table S3). Autosomal and mosaic abnormalities in both genders did not exhibit a clear preference for either infertility type, suggesting a heterogeneous phenotypic impact dependent on specific chromosomal regions and breakpoints (44).

Interestingly, CP, traditionally considered benign variants, demonstrated a notable association with primary infertility (45.1%, 101/224; Supplementary Table S2). In males, the proportion of primary infertility was even higher (50.5%, 54/107), and sex chromosome polymorphisms were exclusively observed in males (9/9), all of whom had primary infertility (Supplementary Table S5). This suggests that submicroscopic structural variations or epigenetic alterations involving the Y chromosome may contribute to subtle spermatogenic disruptions, which are not evident through standard karyotyping (10).

Collectively, these results emphasize the importance of personalized cytogenetic evaluations based on infertility type, advocating for detailed screening strategies, particularly in male patients with primary infertility. Stratified cytogenetic interpretation, considering chromosomal subtype, gender, and clinical history, can enhance diagnostic accuracy and guide targeted genetic counseling and ART treatment planning.

Consanguineous marriage has been historically documented in population-based demographic and public health studies from Middle Eastern and Arab countries, including the UAE (45, 46). This study observed no statistically significant differences in the distribution of marital consanguinity across couple-level karyotype groups (normal, CA, CP) among infertile couples undergoing ART in the UAE. As shown in Table 3, the proportion of consanguineous couples was 39.0% (644/1,639) in the normal karyotype group, 34.6% (8/24) among couples with CA, and 45.1% (40/88) among those with polymorphisms. These descriptive findings establish a demographic baseline for the local ART population but do not imply a causal link to the patients’ own cytogenetic status.

These findings suggest that, while consanguinity is a well-recognized risk factor for autosomal recessive conditions and specific inherited disorders, it may have limited or negligible effects on the occurrence of large-scale chromosomal rearrangements detectable by conventional cytogenetic methods. Similar observations have been documented in both regional and international studies, highlighting weak or non-significant correlations between consanguinity and balanced translocations, structural CA, or polymorphic variations (12, 15, 28, 33, 36).

The relatively high baseline prevalence of consanguinity among couples with normal karyotypes highlights the continued cultural acceptance and persistence of consanguineous unions in this region. This emphasizes the importance of comprehensive genetic counseling tailored to regional practices, particularly addressing recessive gene effects that might not be identifiable by routine karyotyping alone. Additional molecular techniques, such as next-generation sequencing or chromosomal microarray analysis, could further clarify potential submicroscopic genetic risks associated with consanguinity, thereby enhancing genetic counseling accuracy and optimizing reproductive health outcomes (47).

In summary, Table 3 confirms that while the direct impact of consanguinity on cytogenetically visible CA appears limited, ongoing surveillance and comprehensive genetic counseling remain crucial, particularly in populations with high consanguinity rates. Because consanguinity was assessed as marital consanguinity within the infertile couple rather than parental consanguinity of the patients, these findings should be interpreted as descriptive and not causal. Shifting from these couple-level demographic variables, the analysis further explores the clinical relationship between chromosomal findings and specific male phenotypic expressions identified through semen analysis.

The present study provides critical insights into the relationship between CA and semen quality parameters among infertile males undergoing ART. A notably robust association was identified between CA and azoospermia, which was present in 59.18% (29/49) of males with CA (as shown in Table 6). This striking prevalence contrasts sharply with the complete absence of azoospermia in males with normal karyotypes (0%, 0/2,037) and a minimal occurrence among males with CP (2.11%, 2/95). These findings were highly statistically significant (*P* < 0.0001 for normal vs. abnormality; *P* = 0.002 for normal vs. polymorphism, Table 6), underscoring the significant cytogenetic etiology underlying azoospermia cases (10, 15). The predominance of the 47,XXY karyotype among azoospermic patients aligns with established evidence on Klinefelter syndrome, a condition characterized by primary testicular failure and spermatogenic arrest, thus emphasizing the critical role of sex chromosome anomalies in severe male infertility (47). Such findings advocate for the routine use of cytogenetic testing as an integral part of the diagnostic workflow for male infertility, particularly in cases of azoospermia.

From an epidemiological perspective, the overall prevalence of semen abnormalities in our cohort aligns with regional trends but displays distinct local variations. Our reported teratospermia rate (53.42%) is highly consistent with a large-scale UAE study by Omolaoye et al. (2024), which found that approximately 58% of UAE national samples exhibited teratozoospermia (48). However, our findings differ notably from Qatar-based data, where Elbardisi et al. (2018) reported a higher prevalence of azoospermia (6.05%) and oligospermia (23.3%) compared to our cohort's lower rates of 1.42% and 0.05% for the same parameters, respectively (41). These differences may reflect variations in patient recruitment at tertiary referral centers or the impact of regional lifestyle and genetic factors identified in other Gulf-based studies. This comparison emphasizes that while teratospermia is a predominant feature of male factor infertility in the UAE, the prevalence of azoospermia may be lower in this specific patient base than in neighboring populations (41).

Beyond these broad epidemiological trends, Table 6 demonstrates a robust analytical linkage between cytogenetic status and severe phenotypic expression. Our observation that azoospermia is most heavily concentrated within the CA group provides a localized resolution to the 13.9% CA prevalence previously reported among azoospermic Emirati men (12). This phenotypic-cytogenetic correlation mirrors findings in Qatar, where CA were substantially more frequent in patients with azoospermia (10.78%) than in those with severe oligozoospermia (7.5%), reinforcing the clinical priority of genetic workups for non-obstructive azoospermia (9). Collectively, these comparisons suggest that while the UAE patient base presents with a uniquely high burden of morphological impairment (teratospermia), the role of sex chromosome aneuploidies like Klinefelter syndrome remains a consistent and universal driver of severe male factor infertility across the GCC landscape (9, 41).

Other semen quality parameters, including OAT, SOAT, and teratospermia, exhibited less pronounced cytogenetic associations. For instance, OAT was present in 10.20% (5/49) of males with CA compared to 4.47% (91/2,037) in males with normal karyotypes, although this difference was not statistically significant (*P* = 0.0716, Table 6). Despite this apparent trend, statistical analysis suggests a multifactorial or idiopathic origin rather than a strictly cytogenetic cause (44).

Moreover, teratospermia, identified as the most prevalent semen abnormality, occurred in 55.03% (1,121/2,037) of males with normal karyotypes and 46.32% (44/95) of those with CP, further supporting the hypothesis that many sperm morphology defects may not have chromosomal bases but rather involve environmental or other genetic factors not detectable by conventional cytogenetic methods (*P* = 0.0953, Table 6) (33).

Patients with CP showed semen profiles largely comparable to those with normal karyotypes, except for rare cases of azoospermia (2.11%) and slightly elevated frequencies of OAT (5.26%) and SOAT (12.63%). None of these differences reached statistical significance (all *P* > 0.27, Table 6). This reinforces previous suggestions in the literature that polymorphisms, particularly those involving heterochromatic regions, are mostly benign variants with limited or no direct functional impact on fertility outcomes (10, 15).

## 5 Study limitations

This study has certain limitations that should be acknowledged. First, the use of conventional karyotyping for chromosomal analysis may have limited the detection of submicroscopic imbalances, which could be more effectively identified through techniques such as chromosomal microarray analysis (CMA). Second, while CP were identified and documented, their precise functional impact on fertility remains uncertain, necessitating further molecular and functional investigations to clarify their reproductive significance. Third, consanguinity was measured at the couple level (marital consanguinity) rather than parental consanguinity of the patients; therefore, these data cannot directly address whether consanguinity contributed causally to cytogenetic abnormalities observed in the patients.

## 6 Conclusion and future directions

This large-scale cytogenetic analysis of 4,672 infertile patients undergoing ART in the United Arab Emirates reveals that, although the vast majority of individuals exhibit normal karyotypes, clinically important abnormalities, particularly sex-chromosome anomalies, are highly concentrated among males with primary infertility and azoospermia. Mosaic and autosomal abnormalities display broader, more balanced distributions across sexes, whereas sex-chromosome polymorphisms appear exclusively in males with primary infertility, hinting at an under-recognized role in spermatogenic failure. Intriguingly, despite the region's high baseline consanguinity, no statistically significant differences emerged in the distribution of marital consanguinity across couple-level karyotype groups with cytogenetically visible anomalies, suggesting that other genetic or epigenetic mechanisms are driving infertility risk in this population. These findings strengthen the case for routine, sex-specific cytogenetic testing and region-tailored genetic counselling, particularly for men presenting with severe semen abnormalities or primary infertility.

Looking ahead, future work should pivot toward high-resolution genomics, integrating chromosomal microarray analysis with targeted or whole-genome sequencing. to identify submicroscopic rearrangements and gene-level variants that conventional karyotyping overlooks. Prospective, multicenter studies encompassing diverse ethnic groups and stratifying by consanguinity are needed to validate prevalence patterns and clarify genotype, phenotype relationships that may vary across genetic backgrounds. Parallel epigenetic profiling of DNA methylation, histone marks, and small-RNA landscapes, especially in patients exhibiting mosaicism or unexplained infertility, could illuminate how epigenomic dysregulation modulates gametogenesis and embryo competence. Finally, outcome-oriented clinical trials that rigorously evaluate the utility of preimplantation genetic testing, expanded carrier screening, and other precision-ART interventions in couples with defined cytogenetic risks will be essential for identifying the most effective, cost-efficient strategies to improve pregnancy and live-birth rates. By uniting comprehensive cytogenetic screening with molecular and epigenetic analytics in well-designed prospective studies, the field can advance toward truly personalized reproductive care, reducing diagnostic uncertainty, refining treatment selection, and ultimately enhancing the prospects of parenthood for affected couples.

## Data Availability

The data supporting the conclusions of this article will be made available by the authors upon reasonable request, in accordance with applicable ethical and regulatory requirements for patient confidentiality.
